# An orally available 4*'*-fluorouridine prodrug inhibits SFTSV and LCMV infection

**DOI:** 10.1128/jvi.01172-25

**Published:** 2025-09-16

**Authors:** Xiaoqin Jian, Tianwen Hu, Huan Xu, Yuxi Wen, Yumin Zhang, Mengwei Xu, Xiaming Jiang, Junyuan Cao, Li Xiang, Jingshan Shen, Guanghui Tian, Gengfu Xiao, Leike Zhang

**Affiliations:** 1 State Key Laboratory of Virology and Biosafety, Wuhan Institute of Virology, Center for Biosafety Mega-Science, Chinese Academy of Sciences 74614, Wuhan, Hubei, China; 2 University of Chinese Academy of Sciences, Beijing, China; 3 Vigonvita Shanghai Co., Ltd., Shanghai, China; 4 Department of Pediatrics, Union Hospital, Tongji Medical College, Huazhong University of Science and Technology 12443, Wuhan, China; 5 Hubei Jiangxia Laboratory, Wuhan, China; 6 State Key Laboratory of Drug Research, Shanghai Institute of Materia Medica, Chinese Academy of Sciences 58298, Shanghai, China; St. Jude Children's Research Hospital, Memphis, Tennessee, USA

**Keywords:** severe fever with thrombocytopenia syndrome virus, lymphocytic choriomeningitis virus, 4*'*-fluorouridine, nucleoside analog, antiviral agent

## Abstract

Bunyaviruses, a subset of segmented negative-sense RNA viruses, include pathogenic species capable of zoonotic transmission to humans via arthropod vectors and rodent hosts. Pathogenic bunyavirus infections can cause severe hemorrhagic fevers and other life-threatening diseases, posing threats to human health and social stability; however, therapeutic strategies for treating bunyavirus infections remain limited. Here, we report that VV251 hydrochloride salt (VV251), an optimized oral prodrug derivative of 4′-fluorouridine (EIDD-2794), exhibits potent efficacy against severe fever with thrombocytopenia syndrome virus (SFTSV) and lymphocytic choriomeningitis virus (LCMV) both *in vitro* and *in vivo*. In various cell lines, VV251 inhibits SFTSV and LCMV with EC_50_ values in the nanomolar to micromolar range. In lethal rodent models, once-daily oral administration of VV251 at low doses (10 mg/kg for SFTSV; 1 mg/kg for LCMV) achieves complete protection (100% survival), matching the efficacy of T-705 at 300 mg/kg. Additional pharmacokinetic analysis indicates that VV251 has favorable absorption and exposure profiles in both Sprague-Dawley rat and cynomolgus monkey models. This study evaluates the antiviral profile of VV251 and supports its further development as a promising therapeutic candidate.

**IMPORTANCE:**

Bunyaviruses encompass numerous highly pathogenic agents that pose significant threats to human health, including the causative agents of Crimean-Congo hemorrhagic fever, Lassa fever, and Rift Valley fever. The World Health Organization has identified Lassa fever as a priority pathogen requiring urgent research and development efforts in emergency contexts, underscoring the critical need for effective oral antiviral therapies to enhance pandemic preparedness. Here, we report that VV251 hydrochloride salt (VV251), an optimized oral prodrug derivative of 4′-fluorouridine (4′-FlU, EIDD-2794), shows significant efficacy against severe fever with thrombocytopenia syndrome virus and lymphocytic choriomeningitis virus infections, with inhibitory activity in cell culture and protective effects in lethal animal models. Building on the established broad-spectrum antiviral activity of 4′-FlU against multiple high-consequence pathogens (including severe acute respiratory syndrome coronavirus 2, respiratory syncytial virus, Lassa virus, and Junin virus), VV251 emerges as a promising next-generation oral antiviral candidate, offering an orally available therapeutic option to combat these formidable pathogens.

## INTRODUCTION

Segmented negative-sense RNA viruses include bunyaviruses, which are classified within the order *Bunyavirales* and exhibit a broad geographical distribution (1, 2). While most bunyaviruses are transmitted through arthropods and rodents, pathogenic bunyaviruses, such as Crimean-Congo hemorrhagic fever virus, severe fever with thrombocytopenia syndrome virus (SFTSV), Hantaviruses, Lassa virus (LASV), and lymphocytic choriomeningitis virus (LCMV), can cross species barriers to infect humans, causing clinical manifestations like leukopenia, high fever, and neurological symptoms, posing serious threats to human health and social stability (1, 3, 4).

Severe fever with thrombocytopenia syndrome (SFTS) is a severe tick-borne zoonotic disease caused by SFTSV infection (5). The first case was reported in China in 2009, with subsequent cases documented in Japan, South Korea, Vietnam, and Thailand (3, 6–9). SFTS manifests as a potentially fatal hemorrhagic fever in humans, with a case fatality rate (CFR) reaching up to 30% (10). The expanding range of the tick reservoir *Haemaphysalis longicornis* facilitates the potential spread of SFTSV infection, increasing the risk of a global SFTS pandemic (11). In 2017, the World Health Organization prioritized SFTS for research and development, underscoring the urgent need to identify effective antivirals and prepare for its potential impact (12).

Currently, treatment options for SFTSV infection remain limited due to the absence of Food and Drug Administration (FDA)-approved drugs or vaccines. Ribavirin, a potential antiviral against SFTSV with half-maximal effective concentration (EC_50_) values ranging from 3.69 to 8.72 µg/mL (13), has been reported to be insufficient for improving the outcomes of SFTS patients and shows no effect on reducing CFR in patients with a viral load exceeding 10^6^ copies/mL (tested at hospital admission) in several retrospective studies (14, 15). T-705 (favipiravir), an anti-influenza drug approved for human use in Japan and China (16), has demonstrated efficacy against SFTSV and LCMV and improves clinical outcomes in SFTS patients; however, the CFR shows no statistically significant improvement, and the risk of teratogenicity is not negligible due to its distinctive mode of action (4, 17, 18). Benidipine hydrochloride and nifedipine, two calcium channel blockers, were found to be effective against SFTSV infection in SFTS patients, but comprehensive clinical trials evaluating their safety and efficacy are lacking (14). Additional small molecules, including 2*'*-fluoro-2*'*-deoxycytidine (19), amodiaquine (20), hexachlorophene, and the NF-κB inhibitor SC75741, have also been reported to be effective against SFTSV *in vitro* (21, 22), but further validation of these potential antivirals is needed.

Owing to its crucial role and structural conservation in viral replication, RNA-dependent RNA polymerase (RdRp) is an appealing antiviral target (23). Consequently, nucleoside analogs represent the most extensive class of small molecule-based antivirals, as they inhibit viral polymerases and exert antiviral functions (24). In recent years, nucleoside analogs such as zidovudine for acquired immune deficiency syndrome, entecavir and sofosbuvir for hepatitis B and hepatitis C, respectively, and acyclovir for herpes simplex have demonstrated efficacy in treatment (25–28). During the COVID-19 pandemic, remdesivir became the first nucleoside analog approved by the FDA for severe acute respiratory syndrome coronavirus 2 (SARS-CoV-2) treatment (29). The aforementioned examples affirm that RdRp is a reliable viral target for drug development.

4′-Fluorouridine (4′-FlU, EIDD-2794), a promising uridine analog, has shown efficacy against multiple viruses, including respiratory syncytial virus (RSV) (30), SARS-CoV-2, avian influenza virus (31), chikungunya virus(32), enteroviruses (33), and SFTSV (12). A recent study also reported its efficacy against highly pathogenic arenaviruses LASV and Junin virus (JUNV) (34), suggesting the broad-spectrum utility of 4′-FlU as an orally available antiviral drug. However, the chemical instability of 4′-FlU hinders its further development, as determined by high-performance liquid chromatography analyses, which revealed the degradation of 4′-FlU in acidic buffers at 30°C within 7 days, potentially limiting its therapeutic utility (35–37). Building on our previous screening of 4′-FlU prodrug candidates (38), we conducted a systematic evaluation of VV251 hydrochloride (VV251), a novel double prodrug derivative of 4′-FlU, for its antiviral activity against both SFTSV and LCMV. VV251 features three acetyl modifications on the ribose moiety and a nicotinoyloxymethyl group at the N3 position of the uracil base, structural optimizations specifically designed to enhance oral bioavailability while maintaining potent antiviral efficacy. Comparative analysis revealed that VV251 exhibits stronger *in vitro* antiviral activity against SFTSV and LCMV infections than T-705, as evidenced by lower EC_50_ values under identical conditions. In addition to cell-based assays, VV251 exhibits potent antiviral activity and safety in lethal rodent models of SFTSV and LCMV infection. This study establishes the antiviral profile of VV251, supporting it as a promising antiviral candidate with demonstrated oral efficacy against lethal SFTSV and LCMV infections in rodent models, which may expand limited therapeutic options for bunyaviruses and reduce the disease burden they cause.

## RESULTS

### *In vitro* antiviral activities of VV251 against SFTSV and LCMV

To investigate the effectiveness of VV251 at the cellular level, we first evaluated the inhibitory activity of VV251 against SFTSV and LCMV in different cell lines, with parallel comparisons to its parent compound 4′-FlU and T-705, the latter of which exhibits documented antiviral activity against both viruses (4, 17).

Cells were pretreated with different concentrations of the test compounds for 1 hour, followed by infection with SFTSV or LCMV at various multiplicities of infection (MOI) based on the growth properties of each cell line. At 48 hours post-infection, viral RNA copies in the cellular supernatant were measured using quantitative real-time PCR (qRT-PCR) to determine the half-maximal effective concentration values.

**Fig 1:**
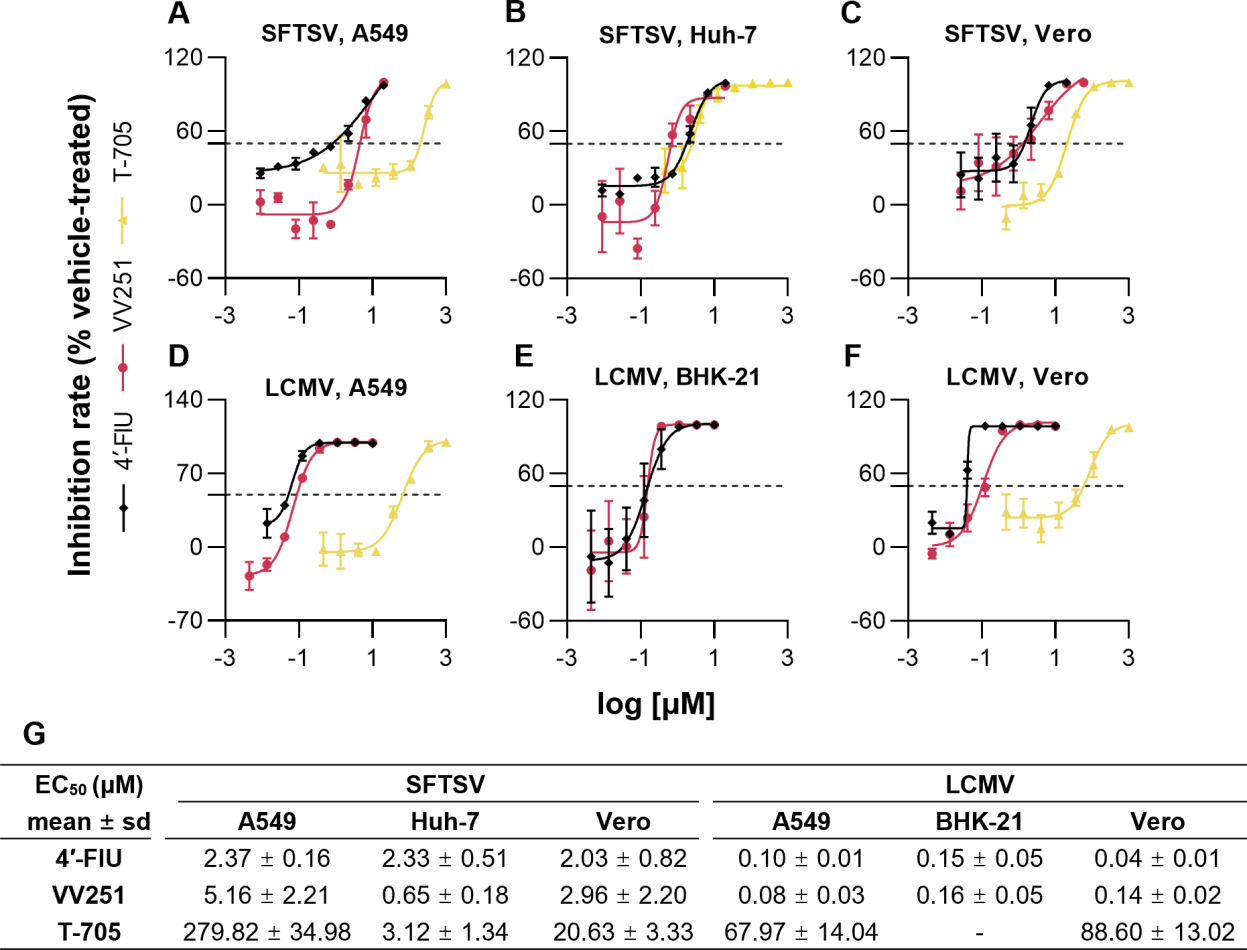
*In vitro* potency of VV251 against SFTSV and LCMV. (**A–F**) Dose-response curves showing the antiviral activity and cytotoxicity of VV251 against SFTSV and LCMV in cell lines. 4′-FlU and T-705 were compared head-to-head against SFTSV in A549 (**A**), Huh-7 (**B**), and Vero (**C**) cells and against LCMV in A549 (**D**), BHK-21 (**E**), and Vero (**F**) cells. Viral RNA copies in the cellular supernatant were measured to determine inhibition rates. Values were normalized to vehicle-treated (dimethyl sulfoxide) controls, and curves were fitted with a nonlinear regression model in GraphPad Prism 9.5.1. (**G**) EC_50_ values of compounds against SFTSV and LCMV determined from viral RNA copy numbers. EC_50_ values were calculated using nonlinear regression in GraphPad Prism 9.5.1. All data are presented as mean ± SD from at least three independent experiments.

The results revealed distinct antiviral efficacy profiles among the tested compounds. VV251 demonstrated potent, broad-spectrum inhibition of both SFTSV and LCMV replication in all cell lines evaluated, with EC_50_ values closely matching those of its parent compound 4′-FlU (range: 0.65–5.16 μM vs 2.03–2.37 μM for SFTSV; 0.08–0.16 μM vs 0.04–0.15 μM for LCMV), consistent with their shared active metabolite 4*'*-fluorouridine triphosphate (4′-FlU-TP) (Fig. 1A through F). Notably, VV251 exhibited better broad-spectrum antiviral activity compared to T-705, demonstrating lower EC_50_ values across all tested cell lines (Fig. 1A through F). Against SFTSV, VV251 showed 54-fold (A549: 5.16 vs 279.82 µM), 4.8-fold (Huh-7: 0.65 vs 3.12 µM), and 7.0-fold (Vero: 2.96 vs 20.63 µM) greater potency than T-705. The potency advantage was even more pronounced against LCMV, with 849.6-fold (A549: 0.08 vs 67.97 µM) and 632.9-fold (Vero: 0.14 vs 88.60 µM) lower EC_50_ values for VV251 (Fig. 1G). These results position VV251 as a substantially more potent antiviral candidate against both bunyaviruses. The efficacy test of T-705 was not performed in BHK-21 cells since it cannot be converted into its ribonucleoside 5*'*-monophosphate by hypoxanthine guanine phosphoribosyl transferase; thus, its ribonucleoside 5*'*-diphosphate and effective active ribonucleoside 5*'*-triphosphate metabolites cannot be formed (39, 40).

Cytotoxicity testing revealed that the half-maximal cytotoxic concentration (CC_50_) values of VV251, 4′-FlU, and T-705 on different cell lines exceeded 500 µM (Fig. S1). Based on EC_50_ values measured by viral RNA copies, these drugs exhibited a wide range of selectivity index (SI, CC_50_/EC_50_). For SFTSV, SIs of VV251, 4′-FlU, and T-705 ranged from 96.94 to 384.25, from 210.67 to 245.98, and from 1.79 to 160.32, respectively. For LCMV, SIs of VV251, 4′-FlU, and T-705 ranged from 3177.56 to 6559.96, from 3389.68 to 12697.20, and from 5.64 to 7.36, respectively (Table S1). The cytotoxicity test of T-705 was not performed in BHK-21 cells for the same reason mentioned for the efficacy test. Taken together, these findings demonstrate VV251’s favorable safety profile, supporting its further investigation as a promising therapeutic candidate.

### VV251 acts as a pyrimidine analog inhibiting SFTSV and LCMV at the post-infection stage

**Fig 2:**
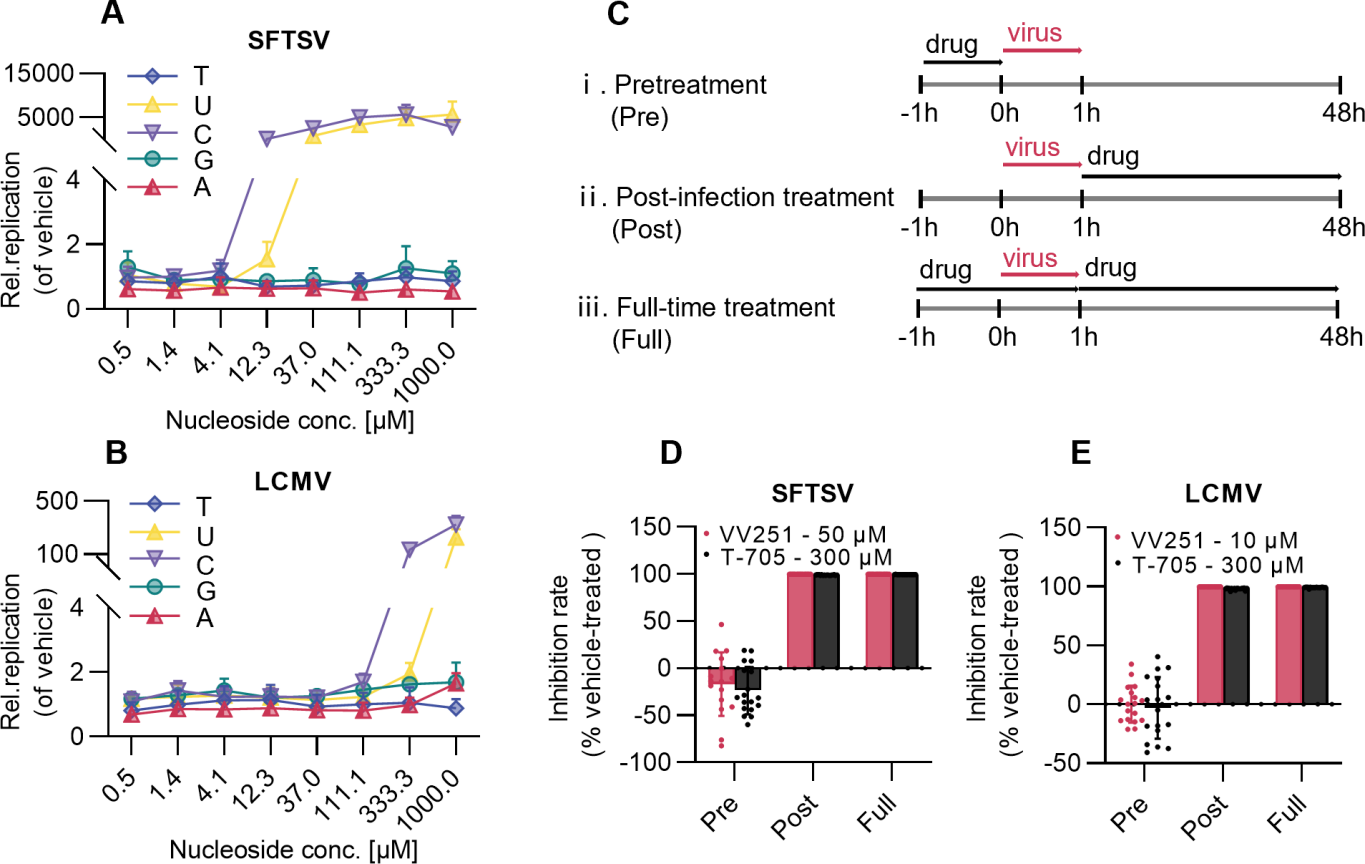
VV251 acts as a pyrimidine analog inhibiting SFTSV and LCMV at the post-infection stage. (**A and B**) *In vitro* nucleoside competition assay. Vero and BHK-21 cells infected with SFTSV (**A**) or LCMV (**B**) were exposed to 10 µM VV251 and increasing concentrations of exogenously added natural nucleosides at infection onset. Values were normalized to vehicle-treated (dimethyl sulfoxide) controls. (**C**) Schematic of time-of-addition experimental design showing three different treatments: (ⅰ) pretreatment of the cell; (ⅱ) post-infection treatment; and (ⅲ) full-time treatment (abbreviated as pre, post, and full, respectively). (**D and E**) Inhibition rates calculated from viral RNA copies of SFTSV (**D**) and LCMV (**E**) after different treatments (described in panel **C**) with VV251. All data are presented as mean ± SD from at least three independent experiments.

To preliminarily investigate the mechanism of action of VV251, we performed a nucleoside competition assay. As the results showed, the antiviral effects of VV251 against both SFTSV and LCMV were competitively reversed by supplementation with exogenous uridine and cytidine (Fig. 2A and B), supporting that VV251 functions as a pyrimidine analog.

To further characterize its antiviral mechanism, we conducted time-of-addition experiments in which SFTSV- or LCMV-infected cells were treated with VV251 according to the schematic (Fig. 2C). T-705 was included as a positive control, given its well-characterized activity as a viral RNA polymerase inhibitor targeting the replication stage (41). Consistent with the positive control T-705, VV251 exhibited potent antiviral activity against both SFTSV and LCMV under post-infection and full-time treatment conditions (Fig. 2D and E). In contrast, pretreatment with VV251 showed minimal to no protective effect, suggesting that its mechanism of action is primarily effective during active viral replication rather than through preemptive viral entry blockade.

### Anabolism and pharmacokinetic profiling of VV251

**Fig 3:**
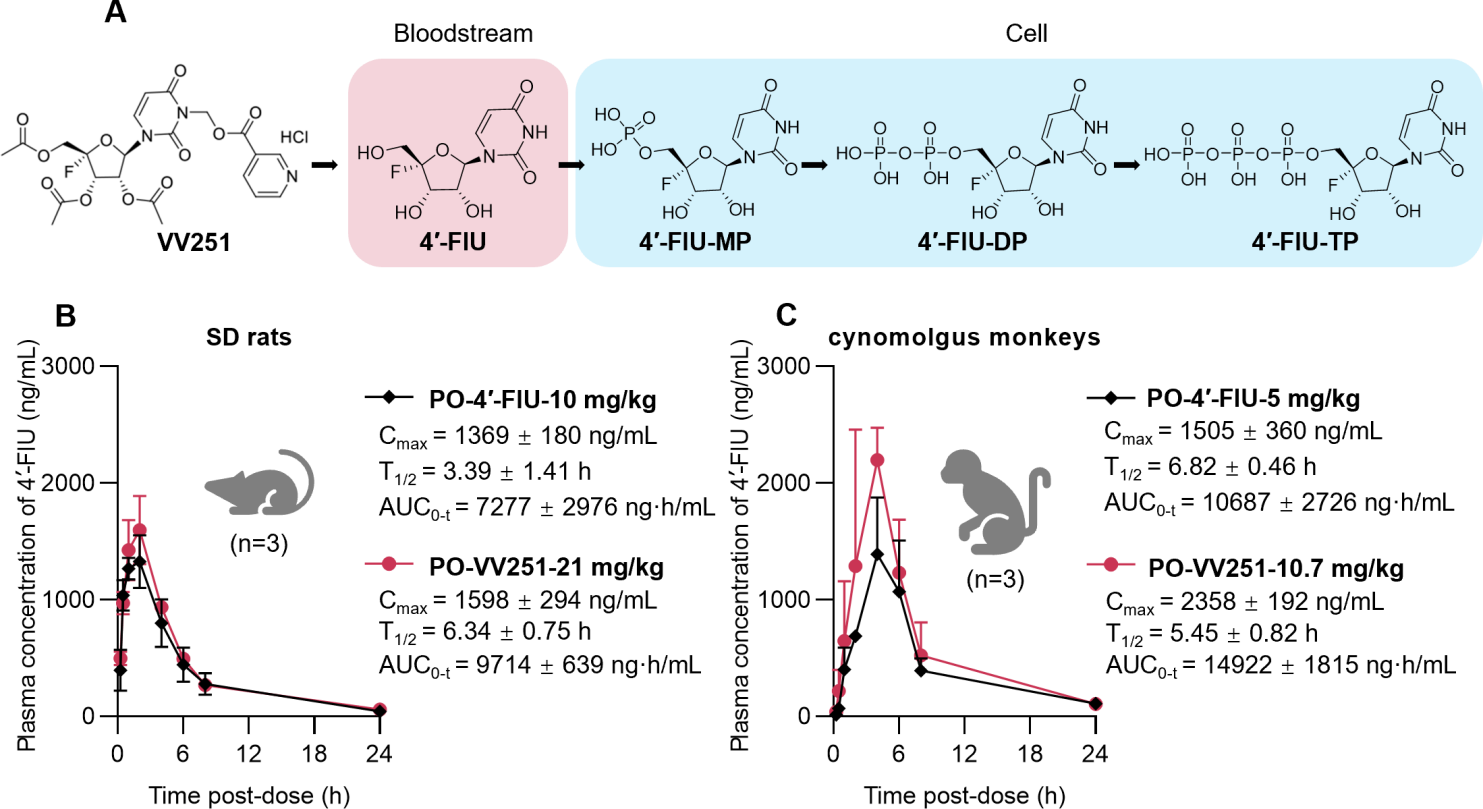
Anabolism sketch and pharmacokinetic profiling of VV251. (**A**) Chemical structures of VV251 hydrochloride salt (VV251) and 4′-FlU with metabolic activation pathway converting VV251 to active NTP metabolite (4′-FlU-TP) after oral administration. (**B and C**) Plasma pharmacokinetic profiles of SD rats and cynomolgus monkeys receiving 4′-FlU or VV251 at indicated doses, pharmacokinetic parameters including half-life (T_1/2_), maximum concentration (C_max_), and area under the curve (AUC) are presented. All data are presented as mean ± SD from at least three independent experiments.

As an ester prodrug, VV251 is rapidly converted to its parent nucleoside 4′-FlU in the intestine and liver by various esterases, which then distributes into the bloodstream and cells and is ultimately metabolized into 4′-FlU-TP via catalysis by a series of kinases (Fig. 3A). Prior to evaluating the *in vivo* antiviral efficacy of VV251, we performed pharmacokinetic (PK) studies in Sprague-Dawley (SD) rats and cynomolgus monkeys following oral administration to compare its systemic exposure with equimolar 4′-FlU and establish appropriate dosing regimens for subsequent efficacy studies. Initial PK studies were conducted in SD rats, with the oral dosing regimen optimized based on our previous experimental findings (38). The PK analysis revealed that oral administration of VV251 (21 mg/kg) achieved comparable systemic exposure to equimolar 4′-FlU, with mean plasma concentrations (C_max_) reaching 1,598 ng/mL and demonstrating a plasma exposure (area under the curve [AUC_0*–t*_]) of 9,714 ng·h/mL. These parameters closely matched those observed for 4′-FlU itself (C_max_: 1,369 ng/mL; AUC_0–*t*_: 7,277 ng·h/mL; Fig. 3B). To further validate these findings in a more clinically predictive model, we extended the investigation to cynomolgus monkeys, which represent the gold standard for nonclinical safety assessment due to their superior pharmacological and metabolic relevance to humans (42). Consistent with the data from SD rats, VV251 (10.7 mg/kg) maintained similar PK characteristics to 4′-FlU (C_max_: 2,358 vs 1,505 ng/mL; AUC_0–*t*_: 14,922 vs 10,687 ng·h/mL; Fig. 3C), confirming its favorable absorption and exposure profiles.

The improved absorption of VV251 compared to 4′-FlU is attributed to its optimized molecular structure. In a previous study, we found that modifying 4′-FlU by attaching three isobutyryl groups to the ribose moiety and a nicotinoyloxymethyl group linked to the imide-nitrogen on the base moiety could improve its chemical stability and enhance its membrane permeability and metabolic stability. Meanwhile, VV251 in its free base form was observed to exhibit favorable stability and PK properties (38). In this study, the hydrochloride salt form of VV251 was selected for PK evaluation, given its favorable aqueous solubility, which may enhance oral bioavailability.

### VV251 is orally efficacious against SFTSV in the A129 model

To evaluate the antiviral potential of VV251 under physiologically relevant conditions, we conducted systematic *in vivo* efficacy studies against SFTSV infection by using IFNAR1 knockout mice (A129) model, which is well known for its hyper-susceptibility to SFTSV infection (12). T-705 was chosen as the positive control due to its reported anti-SFTSV activity in a clinical setting (17).

**Fig 4:**
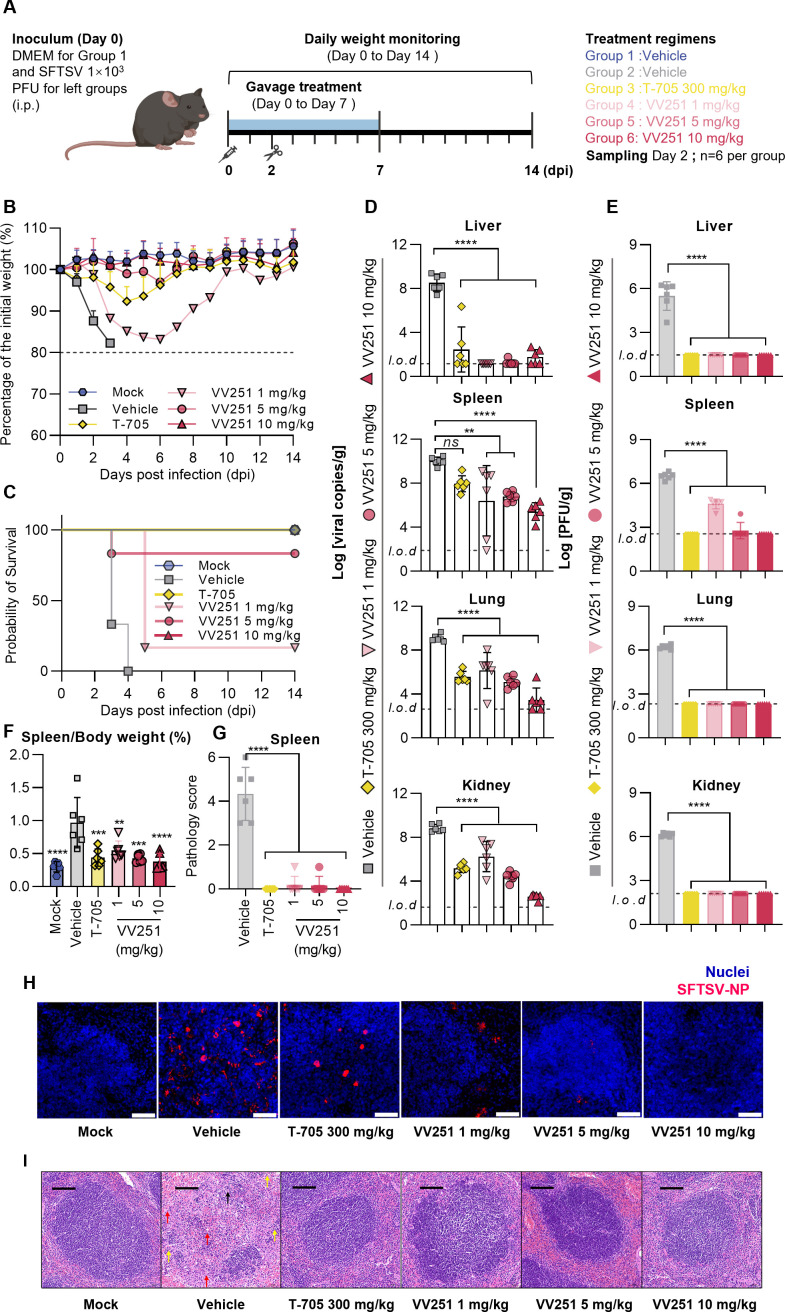
Therapeutic efficacy of VV251 compared with T-705 in A129 mice infected with SFTSV. (**A**) Experimental design for *in vivo* anti-SFTSV efficacy. A129 mice were inoculated with 1 × 10^3^ PFU of SFTSV or DMEM (mock) and orally treated with indicated compounds. Tissues and blood were collected at 2 days post-infection (*n* = 6 per group), while parallel groups were monitored for survival (*n* = 6 per group). (**B**) Daily body weight changes (initial weight = 100%). Mice losing more than 20% of their body weight were considered to have reached the ethical endpoint and were euthanized. (**C**) Kaplan-Meier survival curves. (**D and E**) Viral RNA copies (**D**) and titers (**E**) in organs at 2 days post-infection. (**F**) Spleen-to-body weight ratios. Spleens were harvested and weighed at 2 days post-infection. (**G**) Spleen pathology scores. (**H**) Immunofluorescence staining of spleens. The nuclei were stained with DAPI (blue), the nucleoprotein of SFTSV was stained with Cy3-labeled goat anti-rabbit IgG (H + L) (red). Scale bar: 50 µm. (**I**) Hematoxylin and eosin staining of spleens showing decreased small lymphocytes (black arrow), necrotic cells (red arrow), and neutrophil infiltration (yellow arrow). Scale bar: 100 µm. All data are presented as mean ± SD. Significance was determined via one-way ANOVA compared with the vehicle-treated group, ****P < 0.0001, ***P < 0.001, **P < 0.01.

In the efficacy studies, the mice were intraperitoneally infected with 1 × 10^3^ PFU of SFTSV or an equivalent volume of Dulbecco’s modified Eagle’s medium (DMEM) as mock infection. Subsequently, vehicle, T-705 (300 mg/kg), and VV251 (at doses of 1, 5, and 10 mg/kg) were orally administered via gavage for seven consecutive days in a once-daily regimen starting 1 hour post-infection. The mice were anesthetized and sacrificed 2 days post-infection to obtain organ samples (liver, spleen, lung, and kidney) and blood samples. Additionally, another set of equally treated mice was monitored for changes in body weight and survival throughout the experiment (Fig. 4A).

All animals treated with VV251 at the 10 mg/kg dose and T-705 at the 300 mg/kg dose survived. In the VV251 (10 mg/kg) group, the body weight of the mice remained stable, whereas those in the T-705 group exhibited slight initial weight loss followed by weight recovery at 5 days post-infection (Fig. 4B). Notably, four mice treated with the vehicle and one administered with the intermediate VV251 dose (5 mg/kg) died 3 days post-infection, and the remaining mice in the vehicle-treated group died at 4 days post-infection (Fig. 4C). Moreover, the mice receiving the lowest VV251 dose (1 mg/kg) exhibited prolonged survival compared with those in the vehicle group, but five of them died from infection by day 5, and a significant reduction in body weight was observed, with only one mouse surviving until the end of the study (Fig. 4B and C). Overall, compared with T-705, once-daily treatment with 10 mg/kg of VV251 proved highly efficacious in protecting mice from SFTSV infection, along with maintaining relatively stable body weight.

Compared with vehicle treatment, all doses of VV251 and T-705 led to significant reductions in the virus load in the liver, spleen, lung, and kidney, as assessed via qRT-PCR (Fig. 4D) and immunoplaque assays (Fig. 4E), respectively. Notably, while qRT-PCR analysis revealed no statistically significant reduction of viral RNA copies in the spleen following T-705 treatment compared to vehicle control, immunoplaque assays demonstrated marked decreases in infectious viral titers. This apparent discrepancy likely stems from T-705’s unique mechanism of action as a viral mutagen. Unlike conventional antivirals that directly inhibit viral replication, T-705 induces lethal mutagenesis in viral genomes. This distinct pharmacological action preferentially reduces infectivity rather than viral RNA quantity, explaining the observed dissociation between qRT-PCR measurements (reflecting total viral genetic material) and immunoplaque assay results (measuring functional viral particles) (17).

To gain better insight into the impact of VV251 on mitigating pathogenesis, histopathological analysis of the tissues was conducted. The vehicle-treated group developed pronounced splenomegaly, which was significantly ameliorated by all tested doses of both VV251 and T-705 (Fig. 4F and G). This improvement correlated with substantially reduced histopathology scores (Fig. 4G) and a dose-dependent decrease in SFTSV antigens distribution within the spleen (Fig. 4H). Hematoxylin and eosin (H&E) staining further demonstrated that VV251 and T-705 treatments effectively attenuated SFTSV-induced splenic damage, including loss of white-red pulp demarcation, perivascular leukocyte infiltration, and alveolar septal thickening (Fig. 4I). This protective effect extended to pulmonary tissues, where VV251 and T-705 treatments showed improved histoarchitecture and significantly lower pathology scores (Fig. S2A and B).

Furthermore, ethylenediaminetetraacetic acid (EDTA)-treated whole blood was extracted to assess hematological parameters and the plasma cytokine profile, as hemorrhagic fever can lead to a dysregulated inflammatory response and cytokine storms (43). The viremia caused by infection led to severe leukopenia, lymphocytopenia, and a reduction in the level of platelet count, which were all relieved by T-705 and VV251 treatment (Fig. S3A through D). Notably, the concentrations of interferon-γ (IFN-γ), tumor necrosis factor-α, interleukin-1β, and interleukin-6 rapidly increased in the plasma after viral infection in the vehicle-treated group, whereas treatment with all dosages of VV251 and T-705 significantly reduced the levels of these cytokines, potentially effectively preventing the formation of cytokine storms (Fig. S3E through H).

The results validated the effectiveness of VV251 against SFTSV, as demonstrated by decreased viral load, improved histopathology, reduced viremia, and attenuated proinflammatory responses. Furthermore, once-daily oral administration of VV251 at a dosage of 10 mg/kg in mice showed comparable efficacy and safety to that of T-705 at a dosage of 300 mg/kg.

### VV251 is effective against LCMV in the C57BL/6-*Prf1^tm1Sdz^*/J model

**Fig 5:**
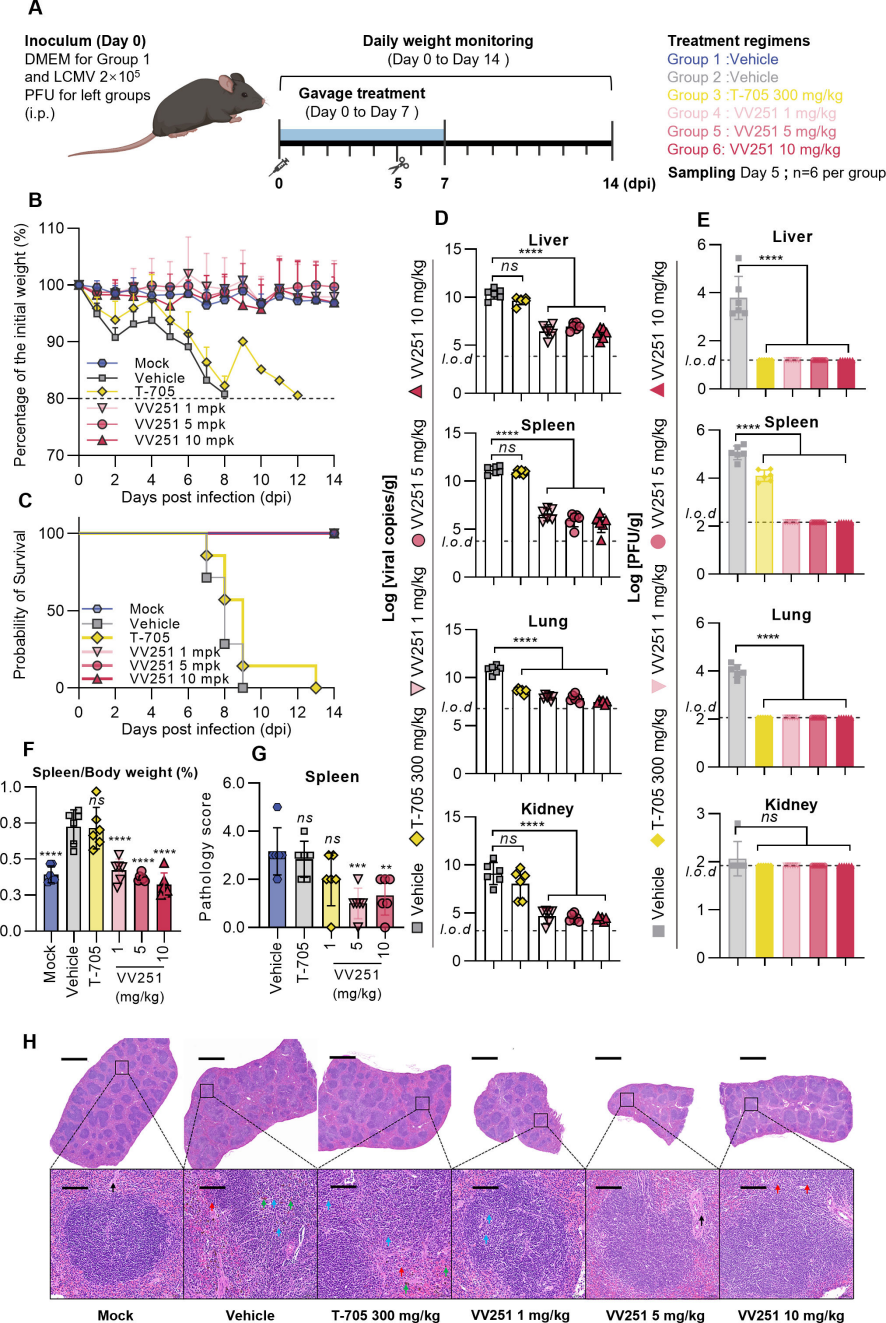
Therapeutic efficacy of VV251 compared with T-705 in C57BL/6-*Prf1*^tm1Sdz^/J mice infected with LCMV. (**A**) Experimental design for *in vivo* anti-LCMV efficacy. C57BL/6-*Prf1*^tm1Sdz^/J mice were inoculated with 2 × 10^5^ PFU LCMV or an equal volume of DMEM (mock) and treated with indicated compounds. Tissues and blood were collected at 5 days post-infection (*n* = 6 per group), while parallel groups were monitored for survival (*n* = 6 per group). (**B**) Daily body weight changes (initial weight = 100%). Mice losing more than 20% of their body weight were considered to have reached the ethical endpoint and were euthanized. (**C**) Kaplan-Meier survival curves. (**D and E**) Viral RNA copies (**D**) and titers (**E**) in organs at 5 days post-infection. (**F**) Spleen-to-body weight ratios. Spleens were harvested and weighed at 5 days post-infection. (**G**) Spleen pathology scores. (**H**) H&E staining of spleens showing scattered lymphocytes (red arrow), increased large lymphocytes (blue arrow), hemosiderin deposition (green arrow), and trabeculae (black arrow). Scale bar: 1,000 µm (top), 100 µm (bottom). All data are presented as mean ± SD. Significance was determined via one-way ANOVA compared with the vehicle-treated group, ****P < 0.0001; ***P < 0.001; **P < 0.01; *ns*, not significant.

To establish the broad-spectrum antiviral potency of VV251, we evaluated its efficacy against LCMV in a C57BL/6-*Prf1^tm1Sdz^*/J mice model, which is known for effective LCMV infection establishment (44). The mice were either infected with 2 × 10^5^ PFU LCMV or received an equal volume of DMEM as mock infection. Following this, they underwent gavage administration of either the vehicle, T-705 (300 mg/kg), or VV251 (at doses of 1, 5, and 10 mg/kg) starting 1 hour post-infection, once daily for seven consecutive doses. At 5 days post-infection, organs (liver, spleen, lung, and kidney) and blood samples were extracted from equally treated sets of animals for detection (Fig. 5A).

Compared with those in the mock group, the body weights of the mice treated with all doses of VV251 remained stable (Fig. 5B). Notably, even the lowest dose of VV251 (1 mg/kg) ensured the survival of the mice. In contrast, all of the mice in the vehicle group succumbed to the infection within 9 days post-infection (Fig. 5C). Compared with VV251, T-705 at 300 mg/kg provided less protection against LCMV infection, as evidenced by the parallel body weight with that of the vehicle-treated group, the early mortality starting at 7 days post-infection, and no survivors remaining by the end of the study (Fig. 5C).

Viral RNA copies and titers in the liver, spleen, lung, and kidney obtained on day 5 post-infection were assessed via qRT-PCR (Fig. 5D) and immunoplaque assay (Fig. 5E), respectively. The findings revealed that VV251 treatment significantly decreased both the number of viral RNA copies and the titer in the liver, spleen, lung, and kidney, and the number of viral RNA copies was more than one order of magnitude lower than that in the vehicle group (Fig. 5D and E). In contrast, T-705 administration reduced the viral titer in the tissues, whereas no reduction in the number of viral RNA copies was observed except in the lungs, probably due to the T-705 unique mechanism of action (Fig. 5D and E).

Additionally, VV251 treatment ameliorated spleen damage efficiently, as evidenced by the normal size of the spleen and lower pathology score (Fig. 5F and G). H&E staining also revealed a clear demarcation between the white and red pulps and the preservation of the marginal zone in the spleen in the VV251-treated groups, whereas the vehicle- and T-705-treated groups presented marked pathological lesions (Fig. 5H).

The analysis of hematological parameters in EDTA-treated whole blood indicated that the oral administration of VV251 ameliorated viremia induced by LCMV infection. In contrast, the white blood cell, platelet, and lymphocyte count of the T-705-treated group were similar to those of the vehicle-treated group (Fig. S4A through D). Additionally, we conducted an analysis of plasma cytokine levels, considering the potential role of cytokine storms in LCMV pathogenicity. Compared with vehicle treatment, treatment with VV251 at all dosages led to a statistically significant reduction in the expression of IFN-γ and granulocyte colony-stimulating factor, whereas T-705 treatment at 300 mg/kg had no significant effect (Fig. S4E and F). Therefore, the reduced cytokine response may be a crucial factor contributing to the protective effect of VV251 treatment against lethal LCMV infection.

On the basis of the preceding results, VV251 at the lowest dose of 1 mg/kg has more potent anti-LCMV effects than T-705 at 300 mg/kg does, as it can protect C57BL/6-*Prf1^tm1Sdz^*/J model mice from lethal LCMV infection while maintaining their body weight and survival rate. VV251 treatment not only reduced the viral load after infection but also induced altered histopathological changes in multiple organs.

## DISCUSSION

Due to the lack of FDA-approved vaccines or specific drugs, clinical treatments for bunyavirus infections are limited, threatening socioeconomic stability and human health. Effective oral antiviral agents are needed to treat outpatients with risk factors for developing severe symptoms. Recently, the uracil analog 4′-FlU has been reported to effectively inhibit Heartland virus (45), SFTSV (12), LASV, and JUNV (34), which all belong to the order *Bunyavirales*. However, further studies demonstrated that 4′-FlU exhibits poor chemical stability in aqueous solutions, especially under acidic conditions (pH <4)(37, 46), likely due to the glycosidic bond’s increased susceptibility to cleavage in such acidic environments, thus hindering its further development as a viable drug candidate.

To address these limitations, we developed VV251, an orally bioavailable prodrug and structurally optimized pyrimidine nucleoside analog of 4′-FlU, designed to enhance systemic delivery of the parent nucleoside following oral administration. Our preliminary investigations revealed that VV251’s free base form demonstrated better stability and pharmacokinetic profiles compared to 4′-FlU, whereas crystallization difficulties and formulation stability concerns precluded its advancement as a lead candidate (38). Drawing upon the successful structural optimization approach employed in VV116’s development (47), we subsequently prepared VV251 hydrochloride salt, which exhibited improved solid-state characteristics and aqueous solubility. These favorable physicochemical properties qualified the hydrochloride salt as an alternative development candidate, prompting further evaluation of its pharmacodynamic properties.

In this study, we used VV251 hydrochloride salt to comprehensively evaluate its antiviral profile, demonstrating its broad-spectrum activity against both SFTSV and LCMV in diverse cell culture systems. PK characterization in SD rats and cynomolgus monkeys revealed VV251’s favorable absorption profile and systemic exposure compared to equimolar 4′-FlU, establishing an optimized dosing regimen for efficacy studies. Using T-705 as a benchmark antiviral agent with established efficacy against both viruses (4, 17), we evaluated VV251’s *in vivo* therapeutic potential. To our surprise, once-daily oral administration of VV251 at lower doses (10 mg/kg for SFTSV; 1 mg/kg for LCMV) achieved complete protection (100% survival), matching the efficacy of T-705 at 300 mg/kg. Notably, VV251 treatment conferred better protective effects against LCMV infection compared to T-705, as evidenced by better maintenance of body weight and attenuated histopathological damage at lower doses.

For optimal pandemic preparedness, antiviral drugs targeting highly conserved viral proteins, particularly RNA-dependent RNA polymerase, offer distinct advantages due to their potential efficacy against emerging viral threats. The strategy of employing nucleoside analogs to inhibit viral RdRp has been successfully utilized for over three decades, with well-documented clinical success (48–50). Our findings demonstrate that VV251, building upon the well-documented antiviral efficacy of its parent compound 4′-FlU, shows promising potential for treating bunyavirus infections but may also possess broad-spectrum antiviral activity against other viruses, while dose optimization may be required when extending its application beyond bunyaviruses.

Despite the observed efficacy of VV251 in both *in vitro* and *in vivo* settings, our study still possessed limitations. On the one hand, the breadth of protection was only tested in mice, and additional animal models are needed for overall evaluation. On the other hand, the specific mechanism of action of VV251 requires further investigation since nucleoside analogs can target the viral RdRp to impede viral replication through three primary mechanisms: delayed chain termination (e.g., remdesivir), obligate chain termination (e.g., sofosbuvir), and mutagenicity (e.g., molnupiravir) (24, 51, 52). Previous studies have reported that the incorporation of triphosphorylated 4′-FlU by the RdRps of RSV and SARS-CoV-2 results in sequence-modulated transcriptional stalling (30), and further investigation is needed to explore the mechanism of action of VV251 against SFTSV and LCMV. Furthermore, to provide valuable insights into the molecular basis of VV251, the resistance profile of VV251 against bunyaviruses needs further investigation; however, no evidence of breakthrough variants of JUNV was observed after more than 18 passages under 4′-FlU selective pressure (34), which indicates that VV251 may possess the potential for a high genetic barrier of viral resistance since it functions in the form of a 4′-FlU-TP.

Effective oral antiviral therapies that target conserved viral proteins and have a high barrier to resistance are crucial for maximizing therapeutic efficacy against emerging viruses. Our findings establish VV251 as a potential orally efficacious inhibitor of SFTSV and LCMV, positioning it as a promising candidate for the treatment of bunyavirus infections. Building on the success of 4′-FlU against various highly pathogenic viruses, VV251 holds promise as a broad-spectrum antiviral drug available orally to combat these pathogens.

## MATERIALS AND METHODS

### Cells and viruses

A549, BHK-21, Vero, and Huh-7 cells were maintained in Dulbecco’s modified Eagle’s medium supplemented with 10% fetal bovine serum (FBS) and 1% penicillin-streptomycin antibiotics at 37°C in a humidified 5% CO_2_ incubator. SFTSV (strain HBMC16_human_2015) was obtained from the China Center for General Virus Culture Collection. LCMV (strain Armstrong) was rescued using a T7 polymerase system as previously described (53, 54).

### Compound sourcing

VV251, 4′-FlU, and T-705 were obtained from Vigonvita Shanghai Co., Ltd. For *in vitro* studies, all compounds were initially dissolved in dimethyl sulfoxide (DMSO) and subsequently diluted in maintenance medium. For *in vivo* studies, VV251 and 4′-FIU were formulated in a solution of 5% DMSO, 5% ethanol, 40% PEG400, and 50% saline. T-705 was formulated in 0.4% carboxymethyl cellulose (CMC).

### Determination of antiviral activity *in vitro*

To perform antiviral assays, monolayer cells (A549, Huh-7, and Vero cells for SFTSV infection; A549, BHK-21, and Vero cells for LCMV infection) were plated into 48-well plates overnight. After 16 hours, the medium was replaced with 200 µL of DMEM (2% FBS) per well containing compounds at specific concentrations within several gradients, and the mixture was incubated for 1 hour. Next, the corresponding virus (MOI = 0.1 for SFTSV; MOI = 0.01 for LCMV) was added. At 48 hours post-infection, viral RNA copy numbers in the cellular supernatant were detected to determine the EC_50_ values by using quantitative real-time PCR. The inhibition rates of the compounds were calculated on the basis of the number of viral copies normalized to that of vehicle-treated (DMSO) controls as the following formula:

Inhibition rate = [1 − (compound-treated/DMSO-treated viral RNA copies)] × 100%

The EC_50_ values were calculated using nonlinear regression in Prism 9.5.1 software. The bar indicates the SD from at least three independent experiments.

### RNA extraction and quantitative real-time PCR

RNA was extracted from the cell supernatants with a DNA/RNA Extraction Kit (Vazyme, China), according to the manufacturer’s instructions. qRT-PCR was performed according to the instructions of the HiScript II One Step qRT-PCR SYBR Green Kit (Vazyme, China) to determine the viral RNA copy number.

### Immunoplaque assay

To determine the viral titers of SFTSV or LCMV, Vero and BHK-21 cells were incubated in 24-well plates separately. After 16 hours, the cells were incubated with 200 µL of 10-fold serial dilutions of viral supernatants in serum-free DMEM for 1 hour, after which the supernatants were discarded, and the cells were washed with phosphate buffer saline (PBS) three times. Next, 1 mL of DMEM containing 1.1% sodium CMC and 2% FBS was added to each well. After incubation for 3–4 days, the cells were fixed with 4% paraformaldehyde in PBS overnight at 4°C, permeabilized, and blocked in 5% nonfat milk containing 0.2% Triton X-100 (diluted in PBS) for 1 hour. The cells were subsequently incubated with anti-SFTSV-nucleocapsid protein (NP) serum (1:16,000 dilution) or anti-LCMV-nucleocapsid protein serum (1:300 dilution) for 2 hours at room temperature. The plates were subsequently washed in PBS containing 0.05% Tween 20 three times, followed by incubation with a horseradish peroxidase (HRP)-conjugated anti-rabbit antibody (1:1,000 dilution) for 1 hour at room temperature. The viral plaques were finally stained using an enhanced HRP-DAB Chromogenic Substrate Kit (TIANGEN, PA110), and the viral titers were analyzed.

### Cytotoxicity assay

For cell viability experiment, A549, BHK-21, Vero, and Huh-7 cells were plated in 96-well plates, with each concentration tested in triplicate. Compounds were diluted twofold across eight gradients starting at 500 µM in maintenance medium (DMEM + 2% FBS). After 48 hours of incubation, the supernatants were removed, and 100 µL of diluted Cell Counting Kit-8 (Beyotime) in maintenance medium was added to the cells. Plates were incubated at 37°C for 2 hours, and absorbance was measured at 450 nm using a spectrophotometer (BioTek). Cell viability was then calculated. All compounds were diluted in maintenance medium containing 2% FBS.

### Nucleotide competition experiment

Vero and BHK-21 cells infected with SFTSV or LCMV were exposed to 10 µM VV251 in combination with 0.5–1,000 µM exogenous nucleosides (MedChemExpress) at the onset of infection. An equal volume of DMSO served as a vehicle control. At 48 hours post-infection, RNA was extracted from the cell supernatants, and the number of viral RNA copies was measured using qRT-PCR. The values are expressed relative to the values for the vehicle-treated samples. Three independent experiments were performed.

### Time-of-addition experiment

A time-of-addition assay was performed, using different treatment patterns, which were divided into three groups. Pretreatment of the cells: the cells were pretreated with VV251 or T-705 for 1 hour and incubated with the virus for 1 hour. Then, the virus was removed, and the medium was replaced with fresh medium without compounds. Post-infection treatment: the cells were infected with the virus for 1 hour, and fresh medium containing VV251 or T-705 was added after the removal of the virus. Full-time treatment: the cells were pretreated with VV251 or T-705 for 1 hour, the virus was added for infection for 1 hour, and the medium was replaced with fresh medium containing VV251 or T-705. All of the cell supernatants were collected 47 hours later for detection.

### Pharmacokinetic studies of VV251 in SD rats and cynomolgus monkeys

Pharmacokinetic studies of VV251 and 4′-FlU in SD rats and cynomolgus monkeys were conducted at Function Biomedical Co., Ltd. Six SD rats (*n* = 3 for each group) were randomly allocated into two groups and fasted for 12 hours before receiving an oral dose of VV251 at 21 mg/kg and 4′-FlU at 10 mg/kg. The vehicle for oral administration of the test compounds consisted of 5% DMSO, 5% ethanol, 40% PEG400, and 50% saline. Blood samples were collected at 0.25, 0.5, 1, 2, 4, 6, 8, and 24 hours post-dosing. The samples were collected in EDTA-K2 tubes, gently mixed, and then centrifuged at 4°C and 2,000 × *g* for 10 minutes. After centrifugation, the separated plasma samples were frozen at −70°C until analysis, which was conducted via LC-MS/MS. The method of pharmacokinetic testing in cynomolgus monkeys was similar to that in SD rats, with oral doses of VV251 and 4′-FlU at 10.7 and 5 mg/kg, respectively.

### Studies of the anti-SFTSV efficacy of VV251 in A129 mice

Six- to 8-week-old A129 mice were randomized into six groups: the mock group, the vehicle group, the group receiving 300 mg/kg T-705, and the groups receiving VV251 at 1, 5, and 10 mg/kg. After being anesthetized by isoflurane inhalation, the mice were infected with SFTSV (1 × 10^3^ PFU) diluted in 200 µL of DMEM via intraperitoneal injection, whereas the mock group was injected with an equal volume of DMEM. At 2 days post-infection, tissues (liver, spleen, lung, and kidney) and blood samples were harvested for efficacy determination (*n* = 6 per group), and another set of equally treated mice was monitored for changes in body weight and survival throughout the experiment (*n* = 6 per group). The tissues were divided into two parts, which were either placed in tissue homogenization tubes for viral copy number and titer detection or fixed in 4% paraformaldehyde for H&E and immunofluorescence staining for the detection of the SFTSV antigen (anti-SFTSV-NP serum). The image information was collected using a Pannoramic MIDI system (3DHISTECH, Budapest) and FV1200 confocal microscopy (Olympus). At the end of the study (14 days post-infection or once the mice lost 20% of their body weight), the remaining mice in each group were sacrificed after anesthetization.

### Studies of the anti-LCMV efficacy of VV251 in C57BL/6-*Prf1^tm1Sdz^*/J mice

Six- to 10-week-old C57BL/6-*Prf1^tm1Sdz^*/J mice were randomized into six groups: the mock group, the vehicle group, the group receiving T-705 at 300 mg/kg, and the groups receiving VV251 at 1, 5, and 10 mg/kg. After being anesthetized by isoflurane inhalation, the mice were infected with LCMV (2 × 10^5^ PFU) diluted in 200 µL of DMEM via intraperitoneal injection, whereas the mock group was injected with an equal volume of DMEM. On day 5, tissues (liver, spleen, lung, and kidney) and blood samples were harvested for efficacy determination (*n* = 6 per group), and another set of equally treated mice was monitored for changes in body weight and survival throughout the experiment (*n* = 6 per group). The tissues were divided into two parts, which were either placed in tissue homogenization tubes for viral copy number and titer detection or fixed in 4% paraformaldehyde for H&E staining. At the end of the study (14 days post-infection or once the mice lost 20% of their body weight), the remaining mice in each group were sacrificed after anesthetization.

### Titration of virus from excised organs

Organs for viral copy number and titer detection were weighed and homogenized in tissue homogenization tubes with 1 mL of DMEM. Next, the homogenates were centrifuged at 5,000 rpm for 15 minutes, and the viral supernatants were collected. Viral copy number and titer detection were performed via qRT-PCR and immunoplaque assay, respectively.

### Hematology and cytokine level analysis

Hematological parameters were analyzed using EDTA-treated whole blood with an automatic blood cell analyzer for animal use (Tecom). The cytokine levels in the plasma of the mice in each group were detected via an ABplex Mouse 6-Plex Custom Panel (Abclonal) according to the manufacturer’s instructions.

### Hematoxylin and eosin staining and pathology score

Tissues fixed with 4% paraformaldehyde were embedded in paraffin according to standard procedures. Then, the embedded tissues were sectioned at 4 µm for staining with hematoxylin and eosin. The pathology score of the lungs is based on the infiltration of inflammatory cells in the alveoli and interstitium, thickening of the alveolar wall, alveolar and interstitial bleeding, and fibrin exudation in the alveolar cavity. A pathology score range of 0–3 was used to evaluate the extent of spleen damage, based on the area of splenic necrosis, the number of hemosiderin-containing cells in the field of view, and the dispersion of splenic nodules; while a range of 0–4 was used for lung damage, based on alveolar and interstitial inflammatory cell infiltration, alveolar septal thickening, alveolar and interstitial hemorrhage, and fibrinous exudate in alveolar spaces. Scores for each animal represent the sum of damage degrees in each spleen or lung and are presented as mean ± SD.

### Statistical analysis

Data from the cell and animal studies were compiled in Microsoft Excel and analyzed using GraphPad Prism 9.5.1 software. The data are presented as the mean ± SD. Significance was determined via one-way ANOVA compared with the vehicle-treated group, ****P < 0.0001; ***P < 0.001; **P < 0.01; *P < 0.05; ns, not significant.

## Data Availability

The data that support the findings of this study are available from the corresponding authors upon request.

## SUPPLEMENTAL MATERIAL

Supplemental material jvi.01172-25-s0001.docx Figures S1 to S4 and Table S1.

ASM does not own the copyrights to Supplemental Material that may be linked to, or accessed through, an article. The authors have granted ASM a non-exclusive, world-wide license to publish the Supplemental Material files. Please contact the corresponding author directly for reuse.
