# Supplementary material for: An orally available 4'-fluorouridine prodrug inhibits SFTSV and LCMV infection
Source: J Virol. 2025 Sep 16;99(10):e01172-25. doi: 10.1128/jvi.01172-25 (PMC12548419; doi:10.1128/jvi.01172-25)
Supplement: Supplemental material — Figures S1 to S4 and Table S1. [file jvi.01172-25-s0001.docx]

**An orally available 4'-Fluorouridine prodrug inhibits SFTSV and LCMV infection**

**Supplemental Files**

**FIG S1**. Cytotoxicity of VV251 against SFTSV and LCMV


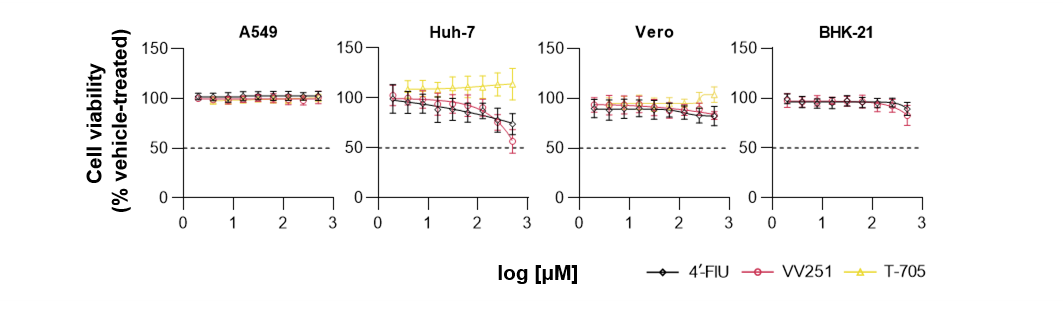


Cytotoxicity of compounds in A549, Huh-7, Vero, and BHK-21 cells were measured by CCK-8 assay. All data are presented as mean ± SD from at least three independent experiments.

**FIG S2**. Histopathological analysis of SFTSV-infected tissues subjected to different treatments


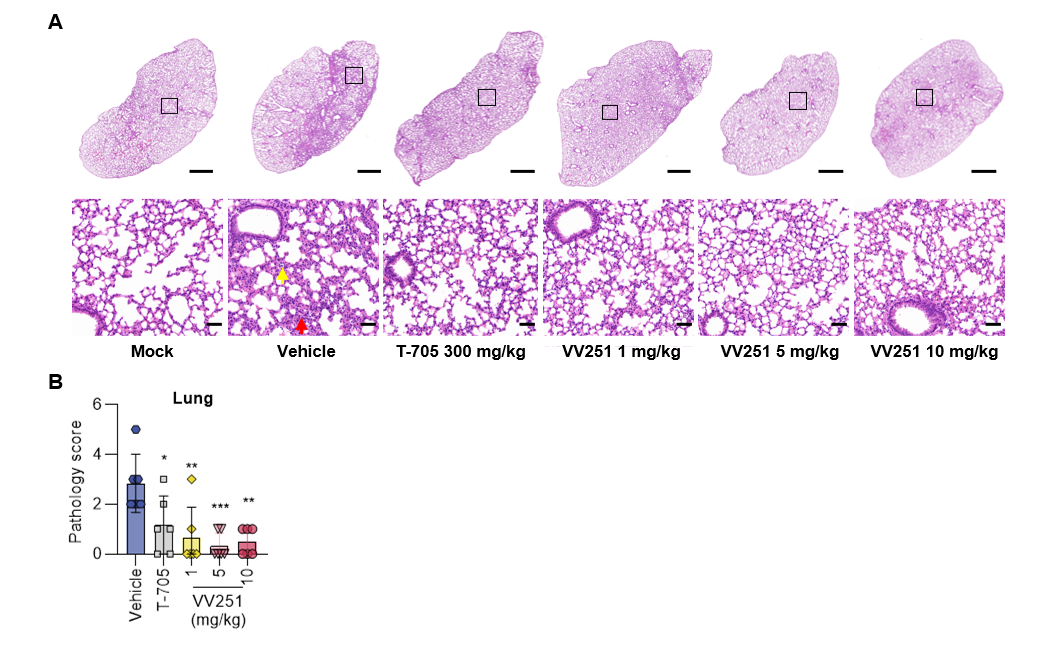


**(A)** H&E staining of lungs showing alveolar wall thickening (red arrow) and inflammatory infiltration (yellow arrow). Scale bar: 1000 μm (top), 50 μm (bottom). **(B)** Lung pathology scores. All data are presented as mean ± SD. Significance was determined via one-way ANOVA compared with the vehicle-treated group, ****P* < 0.001, ***P* < 0.01, **P* < 0.05.

**FIG S3**. Hematological and cytokine profiles in SFTSV-infected mice


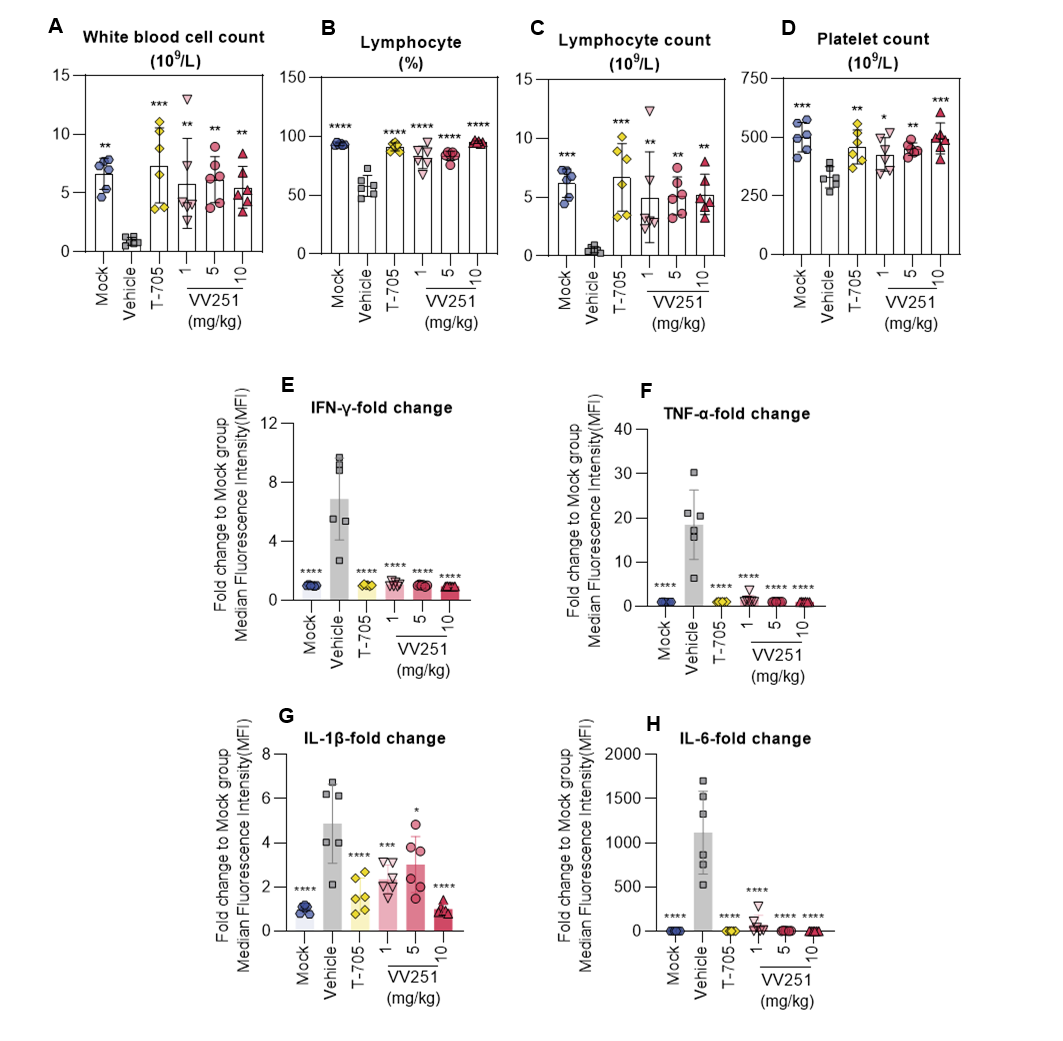


**(A-D)** Hematological examinations of whole blood from mice subjected to different treatments after infection with SFTSV. Including white blood cell count (A), percentage of lymphocytes (B), lymphocyte count (C), and platelet count (D). **(E-H)** Changes in the IFN-γ (E), TNF-α (F), IL-1β (G) and IL-6 (H) levels in the plasma of each group subjected to different treatments after infection with SFTSV relative to the levels in each mock group. All data are presented as mean ± SD. Significance was determined via one-way ANOVA compared with the vehicle-treated group, *****P* < 0.0001, ****P* < 0.001, ***P* < 0.01, **P* < 0.05.

**FIG S4**. Hematological and cytokine profiles in LCMV-infected mice


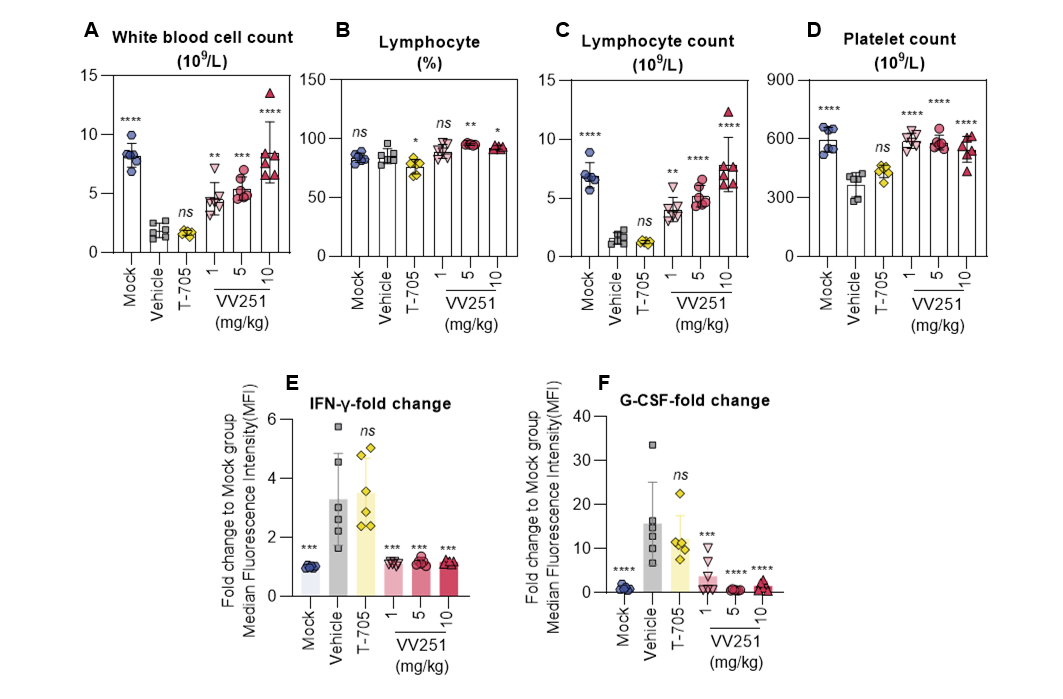


**(A-D)** Hematological examinations of whole blood from mice subjected to different treatments after infection with LCMV. Including white blood cell count (A), percentage of lymphocytes (B), lymphocyte count (C), and platelet count (D). **(E-F)** Changes in the IFN-γ (E) and G-CSF(F) levels in the plasma of each group subjected to different treatments after infection with LCMV relative to the levels in each mock group. All data are presented as mean ± SD. Significance was determined via one-way ANOVA compared with the vehicle-treated group, *****P* < 0.0001, ****P* < 0.001, ***P* < 0.01, **P* < 0.05, *ns*, not significant.

**Table S1**. Selectivity index (SI) values based on CC_50_ and EC_50_

| **SI values** | **SFTSV** | | | **LCMV** | | |
| --- | --- | --- | --- | --- | --- | --- |
|  | **A549** | **Huh-7** | **Vero** | **A549** | **BHK-21** | **Vero** |
| **4'-FlU** | > 210.67 | > 214.23 | > 245.98 | > 4826.57 | > 3389.68 | > 12697.20 |
| **VV251** | > 96.94 | > 384.25 | > 168.75 | > 6559.96 | > 3177.56 | > 3628.85 |
| **T-705** | > 1.79 | > 160.32 | > 24.23 | > 7.36 | - | > 5.64 |
